# The influence of schooling on performance in chess and at the Olympics

**DOI:** 10.1007/s00181-022-02259-9

**Published:** 2022-06-07

**Authors:** David Forrest, J. D. Tena, Carlos Varela-Quintana

**Affiliations:** 1 University of Liverpool Management School, Chatham Street, Liverpool, L69 7ZH UK; 2 Department of Economics, University of Sassari and CRENoS, Via Muroni 25, 07100 Sassari, Italy; 3 Department of Applied Economics, University of Oviedo, Avda. del Cristo s/n, 33006 Oviedo, Spain

**Keywords:** Education capital, Economic resources, Sports economics, Chess, Olympics, I26, J24, L83, Z2

## Abstract

At the macro-level, it is hard to test the hypothesis that increased schooling in a country will raise labour productivity but sectoral analyses may be tractable. In sports, output is homogenous in that countries’ achievements are measurable in the same way. We examine country performances at the Chess Olympiad and the Olympic Games, contrasting tournaments where players in the first use only their minds but most in the second supply substantial physical effort or work with costly physical capital. Modelling success in either leads to a set of results familiar from sports literature: country performance depends on economic resources, represented by population size and per capita income. Supplementary variables capture over-performance by communist/ former communist countries. We then introduce a measure of average years of schooling. This significantly reduces the role of income, especially in chess. It also takes power away from the ‘communist’ variables, especially at the Olympics. These results suggest that much of any effect from income is mediated through schooling: investment in education is associated with elevated productivity. Increased productivity is observed in both settings, one a knowledge-intensive sub-sector and the other dependent to a significant extent on either raw physical strength or expensive capital input.

**Supplementary Information:**

The online version contains supplementary material available at 10.1007/s00181-022-02259-9.

## Introduction

The potential role of educational provision in raising national economic performance has been widely acknowledged since Barro (1991) demonstrated significant correlation between school enrolment-rates and economic growth. However, although decomposition of growth into components attributable to physical and human capital (Henderson and Russell 2005) and also financial development (Badunenko and Romero-Ávila 2013) has been attempted, demonstrating causation at this macro-level is potentially problematic because of the inherent risk of endogeneity given that both a state’s decision to provide schooling opportunities and parents’ decisions to take them up are non-random. Investment in schooling by parents or the state may be undertaken in response to expectations of future growth and may therefore be, to some extent, a leading indicator of rather than a direct cause of enhanced productivity (Bils and Klenow 2000). Further, even where causation is addressed, there is no detailed understanding of the mechanisms by which greater educational provision enhances a country’s performance. Education can improve productivity by providing specific skills or by generating signals which facilitate the identification of more productive workers. However, education may also affect productivity in many other different ways such as, for example, improving individual creativity, strengthening citizenship behaviour and reducing counterproductive performance, such as absenteeism and drug consumption (Ng and Feldman 2009). At the individual level, the acquisition of soft skills appears to contribute significantly to returns from education and it is then plausible to suppose that extending schooling enhances productivity in a variety of settings for reasons beyond those captured by the earning of paper qualifications (Balcar 2017).

Against this background, we investigate the relationship between the stock of schooling embedded in a country’s population and its performance in international sports tournaments. These events present interesting laboratories to test for the relationship between education and productivity at the micro-level. In contrast to productivity in standard industries, which refers to the production of very heterogeneous items using heterogeneous capital inputs, the countries participating in sports tournaments are all attempting to maximise similar ‘output’, for example winners’ medals or ranking points, and playing to the same rules. It should, then, be more straightforward in sport than in many other spheres to test the hypothesis that, *ceteris paribus*, productivity in a sector will be stronger in countries where the population has been exposed to more schooling.

We tested the hypothesis in two contrasting sporting contexts. We first analyse countries’ performances at 13 consecutive editions of the (biennial) Chess Olympiad, a large-scale competition which attracted 180 national teams in 2018. We chose chess because it appears to lie at one extreme of a continuum of sports with different mixes of requirements for cerebral and physical skills. It requires no physical effort from its players, and their productivity is not influenced by differential support from complementary capital goods (equipment, such as tennis rackets or racing yachts, specialist clothing, etc.). In this sense it is more akin to knowledge-intensive industries than to those where physical effort or physical capital are the key inputs.^1^ If schooling raises productivity most in knowledge-intensive settings and if years in school increase productivity by developing cognitive ability, then chess is one sport where the correlation between performance and education should be most clearly observed.

However, cognitive skills, likely to be developed by schooling, will also be relevant, at least to some extent, in most physical sports settings, which demand capabilities such as rapid processing of information and quick and efficient decision taking. For example, controlling for motor skills and sport-specific skills, greater cognitive skills were found to enhance playing performance levels among volleyball (Trecroci et al. 2021) and football (Vestberg et al. 2017) players. Further, there are likely to be other channels through which schooling influences subsequent productivity in chess but which are also relevant for sport generally (and indeed for a wide range of industries). Education promotes and rewards positive attributes such as aspiration, diligence, commitment to a timetable, ability to plan work, allocation of time to promote a future goal, and many other ‘soft’ skills. Again, schools may also deliver training in specific sports skills (including chess skills) because they often encourage participation and give access to clubs, training facilities and coaching.^2^ For all these reasons, we anticipated the possibility of finding a correlation between stock of schooling and performance across sport generally, even if the relationship might not be as strong as in a pure mind sport.

So, in order to provide a point of comparison with chess, we model country medal shares across seven editions of the Summer Olympic Games, held every four years. We chose to study this particular event because it includes a large number and a wide variety of physical sports. Most of these are activities based principally on physical prowess (such as the fighting sports and weightlifting) or investment in superior physical capital (as in equestrian events or sailing) and are therefore more like traditional agriculture or manufacturing than knowledge-based industries.^3^

## Literature review

Among multi-sports events, The Olympics is the focus of unparalleled public interest, with participation by nearly all countries. Further, its prominence is such that it has spawned a significant strand of economics literature which attempts to model the determinants of national team success as measured by country medal shares. This literature provides an empirical framework into which the stock of schooling in the country’s population can be introduced as an additional variable (and indeed two papers on the Olympics, discussed below, have already attempted to do so).

The empirical framework commonly adopted in the literature was set out in an influential paper by Bernard and Busse (2004). Employing a random effects tobit panel estimator, they regressed country medal share (at time t) on the logged values of the country’s population and per capita Gross Domestic Product (GDP) (at time t), adding dummy variables to capture the influence of different political systems and the gain in medal share associated with hosting the Games. All variables were highly statistically significant. But, as almost invariably in econometric analysis, the covariates, here representing population, per capita income and political system, cannot account for all of the patterns observed in the medals tables across Olympics. For example, countries have different preferences in sport and this must account for some of the variation in medal performances. Thus, boxing has high participation in Cuba and it has historically performed well at Olympic Games mainly through winning boxing medals. Its specialism, to the extent that it may have been chosen strategically, is well-chosen because boxing offers many medals across its different weight classifications. By contrast, as noted by Bernard and Busse (2004), there has been limited progress since India has achieved remarkably few medals given its size. This has been speculated to be because the country ‘specialises’ in cricket (which draws to it most athletic talent but has not been in the Olympics since before World War I) and, to a lesser extent, field hockey (where, unlike boxing, the maximum number of medals a country could win is two, one for men, the other for women).

The core theoretical idea underlying the econometric model was that expected medal share depends on the resources available to the country, represented by its population size and income level.^4^ There is a straightforward justification for treating population as a resource for producing success. If innate talent is as if randomly distributed across humanity, larger countries will, on average, have greater endowment of individuals in the right tail of the global distribution, where reside those capable of becoming world class athletes. However, in all sports (including chess), innate ability alone is insufficient for success because individuals’ potential is unlikely to be fulfilled unless they live in a setting where there is, for example, adequate nutrition. Further, physical resources have to be spent on identifying those with the requisite natural talent. This might mean heavy expenditure to encourage and facilitate mass participation. For example, a higher income country is likely to have more swimming pools, allowing high participation in swimming and hence potential swimming champions will be more likely to emerge. (Given that swimming pools outside five-star hotels are all but unknown in most poor countries, it is scarcely surprising that Olympic medals in this sport disproportionately accrue to the wealthiest countries.) Another factor is the high training cost still needed after identifying the most able if they are to be ready for international competition; and resources in the form scientific expertise may have to be devoted to assisting them to this end, for example development of aerodynamically superior clothing for cyclists. In many sports (if not chess), substantial expenditure on venues and other capital items will be needed to allow athletes to practice their discipline to an international standard. For all these reasons, for a given population size, higher average income levels in a country appear likely to be associated with greater sporting achievement by its elite athletes.

Of course similar arguments could apply to other sectors of the economy beyond sport, such as science. To illustrate, German and British nationals have collectively won 53 Nobel prizes in physics but the whole of the African continent has yielded only one winner. We are not inclined to suppose that German and British centres in physics have excelled because there are more naturally able scientists born in those countries than in Africa but rather speculate that centres of excellence can develop much more readily in wealthy countries where the majority of the population has the opportunity to show their talents through extended and well-resourced schooling.

The relationship between income levels and success in sport is confirmed in the empirical models of Bernard and Busse (2004) and subsequent authors. However, the existence of multiple possible channels through which income levels have their effect is left open in the empirical analysis. This is something that we explore when we add a measure of schooling to a model specified in the spirit of Bernard and Busse (2004). If the addition of schooling to the model were to reduce the role of per capita GDP, this would suggest that the effect of income on sporting success was in part mediated through the tendency of richer nations to be able to, and to choose to, provide more schooling. Such a finding would contribute to both the general literature on the societal benefits of education and to the specialist literature on why some nations are more successful than others in the world of sport.

The approach followed in the stream of literature derived from Bernard and Busse (2004) and adapted in the present paper, is based on using only population-wide aggregate/average data to predict performance levels by a country’s elite athletes. But this is not to deny the critical role of specific investment in elite athletes. These types of investment come in many different dimensions, such as high-performance centres, sporting facilities, coaches, nutrition programmes, etc. However, there are at least three reasons for not considering them in the literature to which this paper contributes. First, it is challenging, if not impossible, to measure specific investment in elite sports accurately and across countries. In one of the few attempts to do this in the literature, Forrest et al. (2010) considered an additional covariate, public spending on recreation, which included sports investment as one of its components. They found that adding the new variable improved forecasts of Olympic medals. However, even in this model (and after the inclusion of lagged medals) macroeconomic variables were still relevant, with effects consistent with the previous literature. A second reason for not including information on specific investment in sport is that heavy investment in sport is itself much more feasible for countries with greater economic resources and it is therefore underlying country wealth which might be regarded as the root explanation for why richer countries tend to do better than poorer countries in international sport; ability to afford the best training for the elite is only one of several avenues by which the macro-variable per capita income might account for country achievement levels in elite sport. A final reason to base our analysis on macroeconomic variables is that using physical and mental sport is just a way to test a more general hypothesis that concerns how years of schooling affect the productivity of a homogeneous good. Our results suggest that this beneficial effect would also be relevant to the productivity of other parts of the population.

Prior literature, building on the seminal contribution of Bernard and Busse (2004), has applied their model to account for country success in, for example, later editions of the Games (Scelles et al. 2020), individual sports within the Olympics (Forrest et al. 2017) and international football (Gásquez and Royuela 2016).^5^ Some of the papers in this literature add new covariates to the original model, representing, for example, climatic features of each country or distance to be travelled to the tournament. Only two papers, to our knowledge, consider the relevance of school enrolment-rates to success (in both cases, success at the Olympic Games). Noland and Stahler (2016) include a country’s ‘average years of schooling’ in modelling of medal shares (over two different periods) alongside per capita GDP and report each coefficient estimate strongly significant (though they do not show results from a model without years of schooling and so we were unable to see how introducing it modified results). On the other hand, Krishna and Haglund (2008) found that primary school-enrolment was non-significant in an equation to predict medal share and in a probit equation to model the chance that a country would win a medal at all. A limitation of their work was that they estimated their model over only a single Olympics (that of 2004). Another possible reason for the difference in results from Noland and Stahler (2016) is that they focused on primary education, whereas it could be that only when more formal sports play is offered in later school years do potentially elite athletes have the opportunity to signal their potential.

Prior literature on the economics of chess is scarce and has focused primarily on applications of game theory. However, Minondo (2017) analyses Elo ratings for 146,000 chess players from 106 countries in 2015. Considering levels of achievement reached by players from different federations, he identifies country comparative advantage as a factor driving patterns in levels of achievement of individual players and demonstrates a strong correlation with several proxies for the popularity of the sport by country. The greater the popularity of chess in a country, the greater the probability that its share of innate talent will fulfil its potential because natural ability will be more likely to be exposed to the game (including in schools), more likely to have the opportunity to improve through participation in tournaments and more likely to receive formal training. Croatia is shown to have had the highest productivity in chess as of 2015. Among other jurisdictions exhibiting strong comparative advantage, most were European; but Cuba, Israel and Mongolia also featured. Elo ratings used in the paper emerge from results of individual matches but our different empirical framework is focused on team play. However, our contribution, with a different empirical strategy, should still complement Minondo’s, by identifying sources of comparative advantage. For example, it may suggest that, while the popularity of chess in different countries is the proximate generator of success in the game, it itself is likely to be explained in part by education and economic variables.

## Data

Focusing initially on chess, this section explains the structure of the Chess Olympiad and how we derived values for our dependent variable, intended to capture the degree of success by each national team in each tournament. It then goes on to define our explanatory variables and describe the methods used to construct them.

### The Chess Olympiad

While chess is recognised as a sport by the International Olympic Committee, it has to date not featured in the Games themselves. Consequently, since 1924, an equivalent international chess-specific event has been organised and, since 1950, its periodicity has been every two years. We will analyse data from the ‘Open Section’ of the 13 editions between 1992 and 2016, this span dictated by the availability of data for our focus explanatory variable, schooling. In 2016, the gold, silver and bronze awards at the Olympiad were won by the USA, Ukraine and Russia, respectively; and in 2018 the top country was China.^6^ However, smaller nations have featured in leading positions in the past; for example, Armenia won gold three times over our data period and Israel and Hungary are among the countries to have taken silver.

In the tournament, the federation of each country is permitted to enter one team. An exception is that the host nation is allowed additional teams (1–4 during our period). Teams are also admitted from three non-national federations, representing blind, deaf and physically handicapped players. Teams of four (currently with a fifth, reserve member permitted) each take part in the same number of matches, currently eleven, the schedule following the ‘Swiss Tournament’ format. This structure pairs first-round opponents based on pre-event rankings and, in subsequent rounds, teams play opponents with similar cumulative points. Each match consists of four one-on-one games; two points are earned for winning the match and one point is awarded if the match is drawn (i.e. if each team wins 2 games). Final tournament rankings are determined by total tournament points.

Our dependent variable is the percentage share of the total points in the whole tournament which were won by the particular country at the particular tournament. Points in matches involving the three non-national federations were included in the calculations but, of course, the three teams in question were not included in the regression analysis because they do not carry population, income and schooling data. To illustrate the order of magnitude of our dependent variable, the mean of percentage points share in the 2016 edition of the Olympiad was 0.62, with a standard deviation of 0.21. The top performance in 2016 was 2.26. Every country achieved a positive score and indeed there was only one case of zero points in the 13 editions covered in our analysis.

An alternative and more granular basis for evaluating country performance at the Olympiad would have been the results of individual games within each match. In fact, we also estimated our models with game points share rather than match points share as the dependent variable. However, there was no material difference in findings, so these results are not reported here. Match points share is preferred only because match points are what contribute to final country rankings in the current rules. All calculations of match and game points shares were from results stored at http://www.olimpbase.org

### Population and income data

Data for both population and per capita GDP were sourced from https://data.worldbank.org. Per capita GDP is expressed in 2011 international dollars, calculated using purchasing power parity exchange rates.

Similar to Bernard and Busse (2004), various adjustments had to be made to estimate per capita income for chess countries which did not correspond to countries listed in the World Bank historical data archive. First, as in other sports such as rugby, England, Scotland and Wales compete as separate national teams, while Northern Ireland is joined to the Republic of Ireland in a single federation (in chess two micro-territories in the Channel Islands are also treated as separate entities). In all these cases population was straightforward to obtain but GDP recorded by the World Bank had to be apportioned between component parts of nation states in proportion to gross valued added indices obtained from the UK Office for National Statistics. Second, some countries split during the period, requiring additional calculations to be made, similar to the adjustments made in the cases of England, Scotland, Wales and Ireland. For example, Czechoslovakia was still a single chess federation for the 1992 Olympiad but World Bank GDP data that year were for Slovakia and the Czech Republic as new nation states.

### Schooling

To construct our variable representing exposure of a country’s population to formal education, we exploited time-series maintained by the Institute for Health Metrics and Evaluation (IHME) (healthdata.org), University of Washington. IHME provides annual country estimates of average number of years of schooling completed, for each five-year age/ gender band in the population.^7^ These estimates were derived by IHME from a limited number of censuses and surveys, such that values for many country-years rely on imputation. For example, in a particular country, a decennial census may be the only source of information on educational attainment. However, among those aged over 25, adding to educational attainment is rare and so it is legitimate, for example, to project the mean schooling figure for the 25–29 age group forward to apply to those in the 30–34 age group five years later. This enables many missing values to be filled. The final complete time-series rely on regression analysis where the expected per-capita schooling in a given demographic group in a given country depends on patterns in schooling level by age and gender, and their trends, and on the broad economic region to which the country belongs (Lim et al. 2018).

From these data, we calculated a single figure for the average years of schooling received by members of the current population, deriving weights from population figures by age and gender provided by IHME. Across observations from the 2016 Chess Olympiad, the mean value of the schooling variable was 10.05 years (standard deviation 2.75), with a range from 3.35 to 15.10 years.

**Fig. 1:**
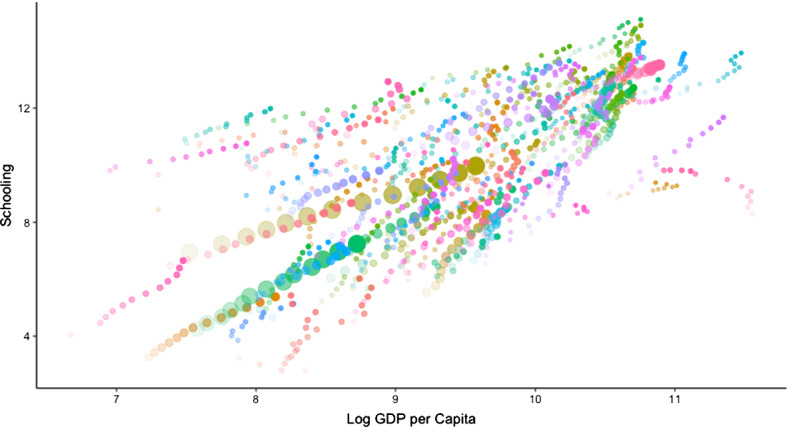
Schooling and per capita GDP

The correlation between the schooling variable and the logged value of per capita GDP was + 0.710. Figure 1, which may be viewed in colour online, presents a scatter plot illustrating the relationship between the two variables. The size of the dots reflects country population size; and different degrees of transparency correspond to different years. To avoid overload, countries with fewer than 10 observations are not shown.

While the scatter plot illustrates clearly that higher income tends to be associated with more extensive schooling, there is nevertheless significant variability. Throughout the period, populations of communist and former communist states tended to have had more schooling on average than would otherwise be expected given their income levels.^8^ When we regressed schooling on log per capita income (with year dummies), the biggest positive outliers in the final year of the period were Kyrgyzstan and Ukraine. The largest negative outliers included small oil-rich states such as Qatar and Bahrain but some large countries (such as Mexico and Pakistan) also had values for schooling appreciably lower than would be predicted from their income levels. Switzerland was an example of a country for which observed and expected years of schooling were close to identical in 2016.

As with the income variable, some ad hoc adjustments were made to account for a few cases where the chess federations did not correspond to nation states identified in the IHME data. For example, for each of England, Scotland and Wales, we used the schooling data for the UK. For Ireland, we used the figures for the Republic even though the jurisdiction of the chess federation extends also to the smaller territory of Northern Ireland.

### Other covariates

We follow Bernard and Busse (2004) and later authors by including dummy variables to represent the political system of countries. They found that communist states typically performed strongly at the Olympic Games. This may be linked to communist regimes choosing to prioritise sport as a means to nation building, promoting physical fitness and securing international prestige (Riordan 2007). Moreover, the central organisation of sport in a planned economy enabled them to direct talent into sport and the lack of political accountability may have permitted them to invest more in the sector than electorates would choose.

Since our data post-date the end of the Cold War, it was appropriate to adapt the specification of the relevant dummy variables adopted by Bernard and Busses (2004) in the analysis of earlier Games, taking into account the changed international landscape, particularly the dissolution of the Soviet Union and the Warsaw Pact. We allocated all countries in the data set which had been part of either the Soviet Union or its sphere of influence to one of two groups, distinguished by whether they had become members of the European Union (EU).^9^ Several of those which are not members of the EU remain with somewhat authoritarian systems of government where it will still be possible for government to direct unusual levels of support to sport regardless of the preferences of their populations. On the other hand, even those which have signalled their commitment to looking westwards may still over-perform because of strong legacy infrastructure. Hence, we anticipated positive coefficient estimates on each of these dummy variables though were prepared for the magnitudes to be different. Additional dummy variables describe a small group of ‘socialist states’ (which, because of missing data issues, proved, by the time of modelling, to include only Vietnam^10^). China has its own dummy variable in recognition of its distinctive style of governance.

Again following prior literature, we include a dummy variable set equal to 1 where the subject country is hosting the Olympiad. A specific reason for its inclusion in the case of chess is that, uniquely, the host is permitted to field more than one team. Here, ‘host’ is entered alongside ‘number of additional teams’, which controls for the host country having the opportunity to secure greater points share simply from playing more matches than other countries. For example, Azerbaijan fielded three teams in 2016, securing more than twice the points share of the gold medal country; but this did not reflect a dominant performance as its ‘A-team’ finished only twelfth in the standings.

### Missing data

Altogether, we had to omit 207 country-year observations from the data to be analysed because of missing information on income, schooling or population. Sometimes a country was left completely out of the analysis because data were never available. In a few cases, it was only omitted for one or two of the years. The large majority of missing observations relate to micro-jurisdictions such as Guernsey, Liechtenstein, Macau and US Virgin Islands. But Cuba was an important country where we had data from only one year; Taiwan had data for only six. Poland had two years where data were incomplete.

Taking into account these omissions, we were left with 1519 country-year observations to be included in our modelling exercise. We attempted, where possible, to increase this number by employing an alternative source of GDP data. Comparable data to that held by the World Bank has been compiled by the Maddison Historical Statistics Project, based at the University of Groningen (https://www.rug.nl/ggdc/historicaldevelopment/maddison). We used Series 2 of its per capita GDP listings, which cover some countries not present in the World Bank data. For country-years in both data sets, we regressed World Bank per capita GDP on Maddison per capita GDP (each measured in 2011 dollars at purchasing power parity) and used the estimate of this regression equation to impute values for country-years absent from the World Bank data.^11^ This allowed us to add 46 country-years to the sample and now only very small jurisdictions accounted for the residual missing observations. All modelling reported below was repeated with the expanded sample but we do not present the results because, with one exception (the result on ‘socialist states’) to be noted below, they were minimally different from those obtained from the sample without imputed values.

## Results (Chess Olympiad)

Table 1 presents regression results from a random effects model.^12^ Note that Bernard and Busse (2004) used a tobit model, to account for the invariably large number of countries which win zero medals at the Olympics. But tobit is unnecessary here because we are modelling points shares at the Chess Olympiad and there is only a single zero among the 1519 observations.

**Table 1:** Regression results, panel data random effects model

	(1)	(2)	(3)	(4)	(5)
ln (population)	0.0568***	0.0524***	0.0531***	0.0549***	0.0552***
	(0.0090)	(0.0067)	(0.0068)	(0.0056)	(0.0050)
ln (per capita GDP)	0.0705***	0.0614***	0.0590***	0.0677***	0.0198*
	(0.0114)	(0.0099)	(0.0102)	(0.0078)	(0.0108)
Host		1.7280***	− 0.0241	− 0.0235	− 0.0249
		(0.2261)	(0.1032)	(0.1047)	(0.1041)
Number of additional teams			0.8484***	0.8471***	0.8478***
			(0.0459)	(0.0461)	(0.0479)
Ex-Soviet bloc (outside EU)				0.2604***	0.1721***
				(0.0197)	(0.0230)
Ex-Soviet Bloc (EU member)				0.2250***	0.1462***
				(0.0210)	(0.0234)
Socialist state (China excluded)				0.1295***	0.0935***
				(0.0144)	(0.0164)
China				0.1232***	0.0876***
				(0.0269)	(0.0268)
Schooling					0.0271***
					(0.0045)
Constant	− 0.5828***	− 0.4502***	− 0.4410***	− 0.5965***	− 0.3694***
	(0.2186)	(0.1674)	(0.1678)	(0.1334)	(0.1295)
Observations	1519	1519	1519	1519	1519
Number of countries	158	158	158	158	158
*R*^2^ between	0.545	0.652	0.662	0.832	0.849
*R*^2^ within	0.188	0.700	0.771	0.771	0.775
*R*^2^ overall	0.286	0.625	0.671	0.786	0.803
Rho	0.302	0.498	0.560	0.380	0.351

Dependent variable: Country percentage points share at the Chess Olympiad

All models estimated with year dummies and over the period 1992–2016 (13 tournaments). Robust standard errors in parentheses

A correlation matrix for the variables included in the modelling is provided in the Supplementary Information (Table S4)

***p < 0.01; **p < 0.05; *p < 0.1

The first column gives results from a basic model where the degree of success just depends on the resources available in the country, represented by logged values of population and per capita income. Both variables are highly significant. As in Bernard and Busse (2004), the magnitudes of the coefficient estimates are rather similar. The results from the most basic model therefore imply that it is weight of gross domestic product which drives success, with little difference made according to the relative contributions to GDP of population size and per capita income.

In column (2), a dummy variable is added to capture any advantage from hosting the event. In the Olympics literature, a significant gain in medal share is always indicated, which could come from a combination of the incentive of a host country to invest heavily in athletes’ preparation (because it wishes to showcase the country’s sport) and the phenomenon of home field advantage observed in many sports settings. Column (2) appears to suggest a strong effect in chess also. However, the result is spurious. Hosts in the Chess Olympiad can accumulate more points simply by taking advantage of a concession which allows them to field more than one team. Once the ‘number of additional teams’ is added as a covariate (column (3)), ‘host’ becomes decisively non-significant. Evidently, there is no home advantage in chess. This is unsurprising. The sources of home advantage in sport have been identified as including familiarity with the climate and with idiosyncrasies of local facilities (e.g. field dimensions), biased officiating, and travel fatigue among visiting competitors (Nevill and Holder 1999). In chess, officials have low or no influence on game outcomes, conditions are homogenous across venues, which are indoors, and, in the case of the Olympiad, the eleven matches played by a team are scheduled over eleven consecutive days, so that most games are not played in the aftermath of travel.

Still following Bernard and Busse (2004), we next add dummy variables representing political systems (column (4)). As in their paper on the Olympics, the introduction of political variables raises explanatory power significantly. On average, countries formerly part of the Soviet Union or in its sphere of influence considerably over-perform relative to their population and income levels, to the extent of more than one standard deviation in the dependent variable (as measured from the 2016 data). The point estimate is a little larger for the group of countries which have not joined the EU but the orders of magnitude are similar. To a lesser extent, the results also indicate over-performance by China and by Vietnam (the sole occupant of the ‘socialist state’ category).

Finally, in column (5), we introduce our schooling variable. The coefficient estimate is positive and strongly significant. In its presence, about one-third of the effect size is shed from the dummy variables representing countries formerly in the Soviet Bloc, indicating that a significant part of their over-performance reflects their typically generous provision for schooling (given their income levels). The coefficient estimate on the income variable is sharply lowered (such that a 1% increase in per capita income is predicted now to increase points share by 0.02 percentage points) and becomes only marginally significant, suggesting that effects from per capita GDP are, to a large extent, mediated through the tendency for populations of higher income countries to have had more widespread education.^13^ The effect size of schooling is modest but not trivial. For example, the difference in the 2016 values of the schooling variable for Portugal and for Poland would raise expected percentage points share by 0.10. To take a more extreme example, Poland’s greater stock of schooling compared with Pakistan would account for a difference of 0.21 in percentage points share in 2016, which was equal to a one standard deviation in the share of points at that edition of the competition.^14^

We noted above that, when we increased the number of observations by allowing per capita GDP to be imputed, results barely changed. However, we report one exception, which relates to the ‘socialist state’ dummy. The expansion of the data allowed Cuba to join Vietnam in this category. As a consequence, the coefficient estimates on ‘socialist state’ in the models corresponding to columns (4) and (5) increased by about three-quarters in each case, reflecting that Cuba, even more than Vietnam, was a considerable ‘over-performer’, with part of this over-performance accounted for by the schooling variable.

Thus, it appears that substantial parts of the effects from per capita GDP and from political variables disappear once a schooling variable is included in the specification. This happens because the income and political system variables are positively correlated with what had been an omitted variable, schooling, and were therefore collecting influences from schooling in their respective coefficient estimates. The question arises of whether these correlations were sufficiently strong to raise concern over potential multicollinearity in the richer specification captured in column (5). We examined the variance inflation factors for all the predictor variables in model (5). Except for the host and additional teams variables (which are not focus variables), all variance inflation factors were comfortably below 4, which indicates that multicollinearity does not appear to compromise the interpretation of the results. Further, we re-estimated model (5) but with ln(per capita income) omitted. The coefficient estimate on the schooling variable increased only modestly, from 0.027 to 0.033, indicating that allowing schooling to collect effects from its correlation with income level added relatively little additional power to the result.^15^

Our conclusion from the analysis of performances at the Chess Olympiad is that per capita income plays little direct role in determining the relative outcomes for different countries. Education appears to displace wealth as a driver of success. The marginal role accorded to income is plausible, in our view. Forrest et al. (2017) reported from sport-by-sport modelling of Olympic Games medals, that income had a much more limited impact on performances in sports where there were low resource costs to participation (e.g. wrestling) than in sports with a need for expensive capital inputs (e.g. a horse for equestrian or the protective equipment required for fencing). Chess appears to be an extreme case where equipment and venue costs are minimal given that a chess set could even be home-made and no specialist venue, not even a sports hall, is required. Barriers to participation from limited income would therefore appear to be lower than in most sporting activities. Hence, when we moved to the case of the Olympic Games, we did not necessarily expect to find closely similar results from those for chess, particularly since schooling has more obvious relevance to skills needed in the setting of a mind sport (akin to a knowledge-intensive sector in the wider economy) rather than in a setting where production depends more on physical prowess (as in many individual sports, traditional agriculture, etc.).

## Results (Olympic Games)

Our analysis of medal shares at the Olympic Games is based on data from the seven editions between 1992 and 2016. While the span of years covered is again determined by the availability of the schooling variable, it conveniently coincides with the post-Cold War era, avoiding the need to reformulate political variables. However, we should note that, as an interim measure following its dissolution, 12 countries which had been part of the Soviet Union joined together as the ‘Unified Team’ for the 1992 Games.^16^ We include the Unified Team in 1992 as an observation in the regression. Per capita GDP of the territory represented by this team was calculated as a population-weighted average across all of the countries covered, using World Bank figures for the population and per capita GDP of each of the newly independent states.^17^ Schooling data were complete and again we used a population-weighted average of the countries. Apart from this single example, we discarded all observations for teams which did not represent individual National Olympic Committees. (From time to time, there have been teams for ‘independent athletes’ or refugees, to accommodate athletes who were stateless or where local Olympic Committees had become inactive because of internal or wider societal problems.)^18^

Missing data issues were similar to those for chess and accounted for 175 missing country-year observations. Using imputed values where per capita GDP was unavailable allowed us to recover some of these, increasing the sample size from 1208 to 1256. Cuba and North Korea (with one year still missing) entered the sample when this was done. This time results displayed no material difference between the samples with and without imputation and, on grounds of ‘purity’, we report the latter rather than the former.

Table 2 presents results from estimation of a panel data tobit model with random effects.^19^ The dependent variable is country medal share. Note that this is not transformed to a percentage figure. The difference from chess is because, in chess, values of the dependent variable, if expressed as a proportion, were very low for all countries and scaling facilitated presentation of results. In modelling the Olympics, we follow the same convention as Bernard and Busse (2004), i.e. we model shares rather than percentage shares. Also like them, we use the tobit version of the panel data random effects estimator, because zero medal share is very frequently observed in the data. Note also that comparison of the absolute values of coefficient estimates between Tables 1 and 2 is not meaningful because the indicator of success is different in the two cases and more concentrated in the Olympics. For example, a weak country team in the Chess Olympiad is still very likely to win a positive points share but a weak Olympics team is rather likely to win no medals at all. The structure of the chess tournament also imposes a severe limit on the maximum proportion of points a country could (theoretically) win compared with medal shares of leading countries at the Olympic Games.

**Table 2:** Regression results, Tobit model with random effects

	(1)	(2)	(3)	(4)
ln (population)	0.0081***	0.0080***	0.0073***	0.0072***
	(0.0008)	(0.0008)	(0.0007)	(0.0007)
ln (per capita GDP)	0.0071***	0.0066***	0.0068***	0.0030***
	(0.0010)	(0.0009)	(0.0009)	(0.0011)
Host		0.0165***	0.0165***	0.0162***
		(0.0024)	(0.0024)	(0.0024)
Ex-Soviet Bloc (outside EU)			0.0155***	0.0055
			(0.0033)	(0.0035)
Ex-Soviet Bloc (EU member)			0.0109***	0.0019
			(0.0035)	(0.0036)
Socialist state (China excluded)			− 0.0113	− 0.0122
			(0.0124)	(0.0146)
China			0.0447***	0.0417***
			(0.0164)	(0.0154)
Schooling				0.0029***
				(0.0005)
Constant	− 0.1937***	− 0.1873***	− 0.1810***	− 0.1656***
	(0.0160)	(0.0156)	(0.0153)	(0.0152)
/sigma_u	0.0175***	0.0174***	0.0158***	0.0148***
	(0.0012)	(0.0012)	(0.0011)	(0.0010)
/sigma_e	0.0063***	0.0059***	0.0059***	0.0059***
	(0.0002)	(0.0002)	(0.0002)	(0.0002)
Observations	1208	1208	1208	1208
Number of countries	188	188	188	188
LR test	870.8	885.8	779.6	681.6
Log likelihood	1538	1560	1577	1592

Dependent variable: Country share of medals at the Olympic Games

All models estimated with year dummies and over the period 1992–2016 (7 Games). Observed information matrix (OIM) standard errors in parentheses

A correlation matrix for the variables included in the modelling is provided in the Supplementary Information (Table S8)

***p < 0.01; **p < 0.05; *p < 0.1

The first and second columns present results from the basic model and a model where ‘host’ is added as a covariate. Results are as expected. Column (3) adds political variables. This is the ‘full’ Bernard and Busse (2004) model, with of course political variables adapted to a later world order. It is striking that the coefficient estimates on ‘host’ and the logged values of population and per capita GDP are all similar to those reported by Bernard and Busse (2004) even though there was only limited overlap between the time periods covered by them and here: they modelled data from 1960 to 1996 whereas our sample was from 1992 to 2016 (hence only two editions in common). This suggests a certain stability over time in the process behind the production of medals. Results on political variables are however different in detail. We find highly statistically significant elevation of performance by former Soviet Bloc countries but the effect size is lower than in Bernard and Busse (2004), where the corresponding political variable referred for most of their period to current rather than historic affiliation with the Soviet Union. A new feature revealed in our results is the extreme over-performance of China over this later period. The coefficient estimate indicates predicted over-performance by China to the extent of 43 additional medals (using the total number of medals available to be awarded at the 2016 Games).

The final column of Table 2 reveals how results changed once schooling was added to the model. The over-performance by former members of the Soviet Bloc countries disappears completely. This indicates that the over-performance evident from column (3) is entirely explained by the legacy effects from the high schooling investment made under Communism and continued since. On the other hand, the coefficient estimate on ‘China’ is unchanged. In that country, it is likely that the ability of government to direct resources to achieving success accounts for ‘over-performance’. This over-performance has no evident association with the level of schooling capital in China, which, throughout the period, is very close to the expected level for its level of per capita GDP.

The coefficient estimate on the log of per capita GDP is diminished by more than one-half with schooling added to the model. This is consistent with a high proportion of the advantage conferred by higher income being mediated through additional schooling provision. The effect size is rather large. A one standard deviation increase in schooling (measured according to data for 2016) is predicted to raise the number of medals by 26. However, because the model is a tobit, this estimate is conditional on a country being assumed to be sure of winning more than zero medals. It would be adjusted downwards for countries with a lower predicted probability of securing any medal at all. In most Games less than half the participating teams take home even one medal, so for a large majority of countries, the predicted probability of winning anything would be appreciably below 1. The estimate of 26 is therefore very much an upper-bound estimate.^20^

Qualitatively, the results from modelling national team performances at the Chess Olympiad and at the Olympic Games point in the same direction. In each case, including schooling in the model takes away explanatory power both from the dummy variables for former Soviet Bloc countries and from the income variable. However, while in the case of chess the effects of income come close to disappearing such that any effects appear to be mediated almost entirely through schooling provision, the results for the Olympics still show a residual if limited independent role for income in driving success. This contrast may be linked to the fact that the practice of chess requires minimal resources whereas Olympic sports include several which are very expensive to play in terms of specialist venues or equipment. Country wealth is therefore still relevant alongside schooling.

The mechanisms by which a higher level of schooling in the population leads to success in chess and Olympic tournaments are open to question. In general, it is possible that the development of cognitive ability and the procurement of soft skills, each associated with schooling, exerts its positive influence in sport as it might be expected to do in other economic activities, leading to greater potential productivity later in life, enough in the case of the most naturally gifted to propel them to success in elite sport. But we are also minded that the variance in enrolment-rates across countries is much greater at secondary than at primary level. This leads us to suppose that differences in average years of schooling between countries is most closely related to how many pupils have continued education to the end of or beyond primary school. It is in these later school years that the direct utility of participation in education to future national success is most obvious. Sports and games are most widely played in education settings where there are often facilities, organised teams and instruction available. The longer the population which remains in school beyond the age where formal replaces informal childhood play, the greater the chance that potentially elite players will be revealed before they disappear into the world of work. Further insight into the mechanisms by which education capital feeds into national success might be gained from future research sport-by-sport.

## Robustness tests

We have demonstrated that there is a statistically significant association between the average quantity of schooling received by a country’s population and the degree of success achieved by its national team in international sports tournaments. This is consistent with the claim that investment in schooling promotes higher worker productivity. Moreover, there is an eminently plausible mechanism for connecting schooling to subsequent participation and success in elite sport.

Nevertheless, there are doubtless several potential explanations of the association between schooling capital and national sporting achievement. In this section, we address the possibility that the results from chess are spurious because the game is most deeply rooted in Europe and Europe happens to be more highly educated than other regions: schooling could be serving as a rough proxy for ‘chess tradition’. We also address the possibility that the results for both chess and the Olympics derive their strength from schooling serving as a subtle indicator of development which captures aspects not picked up by per capita GDP. In this case, the direct effects of schooling on productivity in sport might be smaller than they appear.

These two possibilities are addressed in a similar way. We introduce to the models dummy variables representing World regions, using those defined by the World Bank. If the results from chess just derive from the pre-eminence of Europe in the chess tradition, then the presence of regional dummies should diminish or eliminate the influence of schooling. If schooling is serving just as a proxy for development in the Olympics model, rather than significant in its own right, the coefficient estimate on schooling should again be diminished because, for example, sub-Saharan Africa and South Asia are uniformly less developed than Europe and North America.

In our models, we retain the ‘political’ dummies. The two ex-Soviet Bloc groups may be interpreted alternatively as comprising Eastern Europe. The group ‘socialist state’ includes only Vietnam (data being ‘missing’ for the few other cases). China is its own group. These countries are already allocated to dummy variables in modelling. We allocated all other countries to their respective World Bank regions: ‘East Asia & Pacific’; ‘Europe & Central Asia’ (which we call ‘rest of Europe because the political variables already include Eastern Europe and also Asian republics once part of the Soviet Union); ‘Latin America & the Caribbean’; ‘Middle East & North Africa’; ‘North America’; ‘South Asia’; and ‘Sub-Saharan Africa’.^21^ Each of these regions is now represented by a dummy variable, with ‘East Asia & Pacific’ chosen as reference category on grounds of lexicographic ordering.

Results for both the Chess Olympiad and the Olympic Games are displayed in Table 3. In each case, column (1) repeats the final results from earlier sections and may be compared with column (2) where modelling included the additional regional dummy variables.^22^

**Table 3:** Model results with region dummies added

	Chess Olympiad		Olympic games	
	(1)	(2)	(1)	(2)
ln (population)	0.0552***	0.0540***	0.0072***	0.0072***
	(0.0050)	(0.0045)	(0.0007)	(0.0007)
ln (per capita GDP)	0.0198*	0.0082	0.0030***	0.0032***
	(0.0108)	(0.0114)	(0.0011)	(0.0012)
Host	− 0.0249	− 0.0285	0.0162***	0.0162***
	(0.1041)	(0.1042)	(0.0024)	(0.0024)
Number of additional teams	0.8478***	0.8494***		
	(0.0479)	(0.0471)		
Ex-Soviet Bloc (outside EU)	0.1721***	0.2317***	0.0055	0.0054
	(0.0230)	(0.0353)	(0.0035)	(0.0045)
Ex-Soviet Bloc (EU member)	0.1462***	0.2085***	0.0019	0.0018
	(0.0234)	(0.0361)	(0.0036)	(0.0046)
Socialist state (China excluded)	0.0935***	0.1389***	− 0.0122	− 0.0140
	(0.0164)	(0.0296)	(0.0146)	(0.0140)
China	0.0876***	0.1432***	0.0417***	0.0402***
	(0.0268)	(0.0357)	(0.0154)	(0.0148)
Schooling	0.0271***	0.0260***	0.0029***	0.0024***
	(0.0045)	(0.0054)	(0.0005)	(0.0006)
Rest of Europe		0.0994***		− 0.0006
		(0.0348)		(0.0049)
Latin America and the Caribbean		0.0814***		− 0.0011
		(0.0284)		(0.0044)
North Africa and the Middle East		0.0894**		− 0.0070
		(0.0362)		(0.0050)
North America		0.0396		0.0273***
		(0.0500)		(0.0095)
South Asia		0.0731*		− 0.0124*
		(0.0375)		(0.0074)
Sub-Saharan Africa		0.0063		− 0.0006
		(0.0333)		(0.0048)
Constant	− 0.3694***	− 0.2952**	− 0.1656***	− 0.1623***
	(0.1295)	(0.1360)	(0.0152)	(0.0161)
/sigma_u			0.0148***	0.0158***
			(0.0010)	(0.0009)
/sigma_e			0.0059***	0.0059***
			(0.0002)	(0.0002)
Observations	1519	1519	1208	1208
Number of countries	158	158	188	188
*R*^2^ between	0.849	0.866		
*R*^2^ within	0.775	0.775		
*R*^2^ overall	0.803	0.812		
Rho	0.351	0.332		
LR test			681.6	613.5
Log likelihood			1592	1600

All models estimated with year dummies and over the period 1992–2016 (13 chess tournaments, 7 Olympics). Robust/OIM standard errors in parentheses

***p < 0.01; **p < 0.05; *p < 0.1

Schooling is now being required to work harder to show an effect because its significance depends on detecting a relationship between the relative schooling capital of a country within its region and its sporting outcomes: much of the variability in schooling has been washed away by the inclusion of regional dummies. In the event, the size of the coefficient estimate barely changes in the results for the Chess Olympiad and falls in size only a little in the results for the Olympic Games. In both cases, the coefficient estimate remains strongly significant. These results strengthen the case for there being a direct link between schooling capital and national achievement.

Although the results on our focus variables are essentially unchanged, inclusion of region dummies in the modelling is nevertheless revealing. In chess, a clear hierarchy in regional effects is observed. For reasons unaccounted for in the other variables, Eastern Europe appears to excel in chess. The over-performance of China and Vietnam is even starker when taking account of their geographical positions. Western Europe, North Africa, Latin America and perhaps South Asia are generally strong chess regions, compared with East Asia & the Pacific and North America. Such a hierarchy is much less evident in the results for the Olympic Games. Here different cultures around sports and games are probably less relevant because the prizes awarded are for a basket of sports rather than just one. Different regions’ preferences and strengths are therefore catered for. Only the performances of China and North America stand out compared with other regions, once population, income and schooling levels are taken into account. Each of these has its own distinctive process for producing Olympic athletes. China’s sports system is highly controlled by the state, which allocates very substantial resources to its priority goal, which is international success (Zheng et al. 2018). North America, at least in the USA and, to a lesser extent, Canada (Bermuda is the only other country allocated to this region), has the distinctive feature that the tertiary education system funds and trains many athletes on a possible pathway to the Olympic Games, a route unavailable in other areas of the world.

We carried out two further robustness tests. First, in the particular case of the Chess Olympiad, the ‘Open Section’ is, in principle, open to teams including females; but, in practice, the coexistence of a parallel women’s competition at the same venue and to the same format renders it essentially an all-male affair. We therefore re-estimated the model reported in column (2) of Table 3 with the schooling variable now referring to average years of schooling among males of 15 years and older (instead of for the combined male and female population of 15 years and older). The signs and reported patterns of significance remained the same (with some increase in goodness-of-fit). The coefficient estimate on the new schooling variable was + 0.0255, i.e. barely changed. Second, we re-estimated the same model, for both the chess and the Olympics cases, including the logged value rather than the level of schooling, to allow the effect of average years of schooling in a country to exert a multiplicative rather than an additive effect on the measure of achievement in the tournament. Again there were minimal changes to the findings on other covariates, including the GDP variable. The schooling variable remained very strongly significant in the chess equation (with a coefficient estimate of + 0.1283). In the Olympics equation, the coefficient estimate (+ 0.0075) on schooling was now not quite significant at the 1% level but still had a *p*-value of 0.015. Hence, results with this alternative specification would not change any of our conclusions.

## Closing remarks

In identifying countries with comparative advantage in chess, based on individual player ratings, Minondo (2017) identified the sources of advantage as twofold: the number of players drawn to chess and the opportunities to practice and improve chess skills. Given that initiation of chess is likely often to be in a school setting, his conclusion is not inconsistent with our finding that greater exposure to schooling in the population is associated with greater success in the Chess Olympiad. It would appear likely that similar sources of comparative advantage would apply to other sports as well; and we duly found that greater schooling provision also appeared to deliver greater national success at the Olympic Games. In fact, much of the impact of income levels on winning Olympic medals identified in prior literature appears to be attributable to schooling. Even so, at the Olympics, there is still a residual direct role for income in accounting for the distribution of medals. Again though this may feed back to the sources of comparative advantage proposed by Minondo (2017). Some Olympic sports are very expensive for participants. In poor countries, these will attract few players (and resource limitations may prevent their provision in schools); and the cost of facilities may preclude regular practice. Income per se is not so important in chess in terms of the direct costs of play or its ability to fund capital inputs required for successful outcomes.

We have attempted to contribute to two strands of economics literature. First, a theme in macroeconomics is that investment in education pays off for countries by raising the productivity of future workers. But this is hard to test at the macro-level and building up sectoral studies may lead to a greater evidence base. We show that, for the sports sector, exposure to schooling is indeed a predictor of greater performance by individual workers. Moreover we find increased productivity from schooling not only in a cerebral sport but also in a set of sports where the premium might be thought to be on physical labour or capital input. This might indicate that investment in schooling will have its pay-off not only in knowledge-intensive industries but also in sectors where human capital might appear, on the surface, to be less important. It is true that we observe the performance of a country’s very best workers (who qualify for the national team) but they are likely to be at the top of a national competitive hierarchy such that, if they are world class, those immediately below will seldom be mediocre. Second, we have added to the specialist but vigorous literature on what determines national sporting success. In this literature, country per capita income is well-established as a predictor of a country’s standing in international competition. However, the mechanism by which income level has its effect has been little explored. We have demonstrated that a significant part of the effect of income, perhaps all of it in some activities, is mediated through schooling provision, a demonstration at the sector level of the benefits of human capital investment.

## Supplementary Information

Supplementary file 1.

Supplementary file 2.

## Data Availability

The datasets of this paper (1. Code and programs, 2. Data, 3. Detailed readme files) are collected in the electronic supplementary material of this article.

## Footnotes

1 The Organisation for Economic Co-operation and Development (OECD) uses the term knowledge-based industry to refer those industries relatively intensive in their inputs of technology and/or human capital (OECD 1999). Chess is a sport with minimal capital input and no necessity for the employment of technology: human capital is the key input.

2 Chess is even part of the formal school curriculum in Armenia and Hungary (Jerrim et al. 2018).

3 It is of course true that some physical sports in the Games also require strategic thinking. On the other hand, the most obvious cases of this are team sports, such as football and hockey, which collectively account for a small proportion of the medals awarded since there are typically only two events, one for each gender. Individual sports such as swimming and weightlifting have multiple classes and generate a high proportion of medals, the ‘output’ we model.

4 While theory offered no guidance on the form of the production function, their specification was consistent with a Cobb–Douglas.

5 Some of this later work, such as Scelles et al. (2020), was motivated by seeking to forecast medal totals at upcoming Games. To this end, they include a lagged dependent variable, which considerably raises forecasting performance, reflecting that past performance by a team is a good predictor of performance next time. However, our purpose is not to forecast but rather to identify structural characteristics which drive country success. Hence, as in the main results of Bernard and Busse (2004), we deliberately exclude the lagged dependent variable from our modelling.

6 The 2020 Olympiad was forced online by coronavirus. Following a global connectivity outage, the organisers had to declare joint winners, India and Russia.

7 Years of education completed are capped at 18 years of education.

8 For further illustration, descriptive statistics by political system for the data analysed in the Chess Olympiad modelling are shown in the Supplementary Information (Table S1). Corresponding data for the Olympic Games sample are presented in Table S5.

9 East Germany became part of a new unified Germany state, which we do not treat as formerly communist even though some of its territory is.

10 North Korea never competed in the Chess Olympiad over our data period.

11 On average, the World Bank estimate of per capita GDP was 2.1% higher than the Maddison estimate.

12 Descriptive statistics are shown in the Supplementary Information (Tables S2 and S3) together with a correlation matrix for the variables included in modelling (Table S4). We provide descriptives for the final year of the data set as well as for the whole sample as this illustrates differences between countries at a single point of (recent) time.

13 Although it is perhaps surprising that adding a collinear regressor, such as schooling, reduces the estimated standard error for some variables, this is consistent with econometric intuition. The reason is that the standard error of the estimated parameter is a function of the degree of collinearity among the variables and the Mean Square Error (MSE). The variance increases or decreases depending on which effect is more prominent.

14 At the end of our data period, Poland had the twelfth highest value of the schooling variable. Iceland had the highest value.

15 It is possible that the effects of schooling are exaggerated by the results because schooling is correlated with omitted variables which represent underlying factors that contribute to a country’s success. For example, the level of development might matter to country outcomes and not be proxied adequately by per capita income. In robustness tests reported in Sect. 6 below, we will report that results were broadly similar when we introduced dummy variables for regions with different degrees of development, indicating that estimates of schooling effects were unlikely to be explained by schooling’s correlation with stage of development.

16 These twelve comprised all the countries which had been part of the Soviet Union except for the three Baltic states, each of which was represented independently at the Games.

17 GDP figures were not provided for Moldova but its omission is unlikely to be important since it accounted for only 1.3% of the population of the whole territory.

18 In the Olympics, England, Scotland and Wales compete together as Great Britain. The Irish Olympic Committee represents both the Republic and Northern Ireland. However, Northern Irish athletes may compete under either flag. Consequently, for income and schooling variables, we use data for the United Kingdom for the Great Britain team and data for the Republic of Ireland for the Ireland team.

19 Descriptive statistics for the data set are presented in the Supplementary Information (Tables S6 and S7).

20 Multicollinearity appears not to be a significant issue in the final specification. The variance inflation factor was below 4 for every one of the covariates included in the model for which results are displayed in column (4).

21 The only exception to our following World Bank definitions was that we reallocated Malta from ‘Middle East & North Africa’ to ‘Rest of Europe’, in our view a more natural placement given its membership of the European Union.

22 Data on schooling levels by region, for the case of the Chess Olympiad data set are presented in the supplementary information (Table S9).
