# Supplementary material for: The influence of schooling on performance in chess and at the Olympics
Source: Empir Econ. 2022 Jun 7;64(2):959–82. doi: 10.1007/s00181-022-02259-9 (PMC9171080; doi:10.1007/s00181-022-02259-9)
Supplement: Supplementary file 1 [file 181_2022_2259_MOESM1_ESM.docx]

THE INFLUENCE OF SCHOOLING ON PERFORMANCE IN CHESS AND AT THE OLYMPICS

SUPPLEMENTARY INFORMATION

Table S1. Descriptive statistics by political system (Chess Olympiad)

|  | Population  (millions) | GDP per capita, (PPP Dollars) | Schooling  (years) | Obs |
| --- | --- | --- | --- | --- |
|  |  |  |  |  |
| Ex-Soviet Bloc (EU member) | 9.44 | 23,401.90 | 13.04 | 67 |
|  | (10.94) | (4,263.41) | (0.54) |  |
| Ex-Soviet bloc (outside EU) | 16.91 | 9,955.37 | 11.17 | 262 |
|  | (31.50) | (5,966.53) | (1.01) |  |
| Socialist state (China excluded) | 82.86 | 3,438.99 | 7.59 | 13 |
|  | (7.016) | (1,383.69) | (0.71) |  |
| China | 1,286.34 | 6564.560 | 8.46 | 13 |
|  | (67.40) | (4,194.63) | (0.97) |  |
| All other countries | 44.06 | 21,353.05 | 8.99 | 1,164 |
|  | (126.33) | (20,486.39) | (2.95) |  |

Means are shown with Standard deviations in parentheses. GDP per capita is in purchasing power parity (PPP) constant 2011 international dollars.

Table S2. Descriptive statistics (Chess Olympiad analysis)

|  | Obs | Mean | Std. dev. | Min | Max |
| --- | --- | --- | --- | --- | --- |
|  |  |  |  |  |  |
| Percentage points share | 1,519 | 0.763 | 0.293 | 0.000 | 4.615 |
| ln (population) | 1,519 | 16.156 | 1.738 | 10.978 | 21.044 |
| ln (per capita GDP) | 1,519 | 9.379 | 1.068 | 6.231 | 11.698 |
| Host | 1,519 | 0.009 | 0.092 | 0 | 1 |
| Number of additional teams | 1,519 | 0.018 | 0.203 | 0 | 4 |
| Ex-Soviet bloc (outside EU) | 1,519 | 0.172 | 0.378 | 0 | 1 |
| Ex-Soviet Bloc (EU member) | 1,519 | 0.044 | 0.205 | 0 | 1 |
| Socialist state (China excluded) | 1,519 | 0.009 | 0.092 | 0 | 1 |
| China | 1,519 | 0.009 | 0.092 | 0 | 1 |
| Schooling | 1,519 | 9.530 | 2.852 | 1.117 | 15.102 |

Table S3. Descriptive statistics (Chess Olympiad analysis- final year observations)

|  | Obs | Mean | Std. dev. | Min | Max |
| --- | --- | --- | --- | --- | --- |
|  |  |  |  |  |  |
| Percentage points share | 142 | 0.622 | 0.213 | 0.161 | 2.258 |
| ln (population) | 142 | 16.217 | 1.703 | 12.222 | 21.044 |
| ln (per capita GDP) | 142 | 9.513 | 1.066 | 6.690 | 11.648 |
| Host | 142 | 0.007 | 0.084 | 0 | 1 |
| Number of additional teams | 142 | 0.014 | 0.168 | 0 | 2 |
| Ex-Soviet bloc (outside EU) | 142 | 0.113 | 0.317 | 0 | 1 |
| Ex-Soviet Bloc (EU member) | 142 | 0.077 | 0.268 | 0 | 1 |
| Socialist state (China excluded) | 142 | 0.007 | 0.084 | 0 | 1 |
| China | 142 | 0.007 | 0.084 | 0 | 1 |
| Schooling | 142 | 10.046 | 2.751 | 3.347 | 15.102 |

Table S4. Chess Olympiad models: Polychoric correlation matrix

| Variables | (1) | (2) | (3) | (4) | (5) | (6) | (7) | (8) | (9) | (10) |
| --- | --- | --- | --- | --- | --- | --- | --- | --- | --- | --- |
| (1) Percentage points share | 1.000 |  |  |  |  |  |  |  |  |  |
| (2) ln(population) | 0.262 | 1.000 |  |  |  |  |  |  |  |  |
| (3) ln(per capita GDP) | 0.144 | -0.228 | 1.000 |  |  |  |  |  |  |  |
| (4) Host | 0.739 | 0.255 | 0.141 | 1.000 |  |  |  |  |  |  |
| (5) Number of additional teams | 0.399 | 0.263 | 0.144 | 0.901 | 1.000 |  |  |  |  |  |
| (6) Ex-Soviet bloc (outside EU) | 0.476 | -0.139 | -0.260 | 0.315 | 0.332 | 1.000 |  |  |  |  |
| (7) Ex-Soviet Bloc (EU member) | 0.074 | -0.187 | 0.376 | -0.864 | -0.856 | -0.936 | 1.000 |  |  |  |
| (8) Socialist state (China excluded) | 0.082 | 0.479 | -0.459 | -0.827 | -0.819 | -0.925 | -0.864 | 1.000 |  |  |
| (9) China | 0.163 | 0.914 | -0.273 | -0.827 | -0.819 | -0.925 | -0.864 | -0.827 | 1.000 |  |
| (10) Schooling | 0.294 | -0.236 | 0.710 | 0.203 | 0.214 | 0.462 | 0.802 | -0.261 | -0.146 | 1.000 |

Table S5. Descriptive statistics by political system (Olympic Games)

|  | Population  (millions) | GDP per capita, (PPP Dollars) | Schooling  (years) | Obs |
| --- | --- | --- | --- | --- |
|  |  |  |  |  |
| Ex-Soviet Bloc (EU member) | 9.65 | 23427.63 | 13.02 | 39 |
|  | (10.99) | (4516.03) | (0.56) |  |
| Ex-Soviet bloc (outside EU) | 18.04 | 10061.82 | 11.18 | 133 |
|  | (37.99) | (5914.91) | (1.01) |  |
| Socialist state (China excluded) | 44.24 | 3438.58 | 6.55 | 14 |
|  | (40.38) | (1476.27) | (1.35) |  |
| China | 1285.04 | 6697.20 | 8.46 | 7 |
|  | (75.54) | (4657.11) | (1.08) |  |
| All other countries | 31.34 | 15971.46 | 7.69 | 1015 |
|  | (101.35) | (19462.23) | (3.18) |  |

Means are shown with Standard deviations in parentheses. GDP per capita is in purchasing power parity (PPP) constant 2011 international dollars.

Table S6. Descriptive statistics (Olympics analysis)

|  | Obs | Mean | Std. dev. | Min | Max |
| --- | --- | --- | --- | --- | --- |
|  |  |  |  |  |  |
| Share of medals | 1,208 | 0.006 | 0.015 | 0.00 | 0.14 |
| ln (population) | 1,208 | 15.685 | 1.959 | 10.94 | 21.04 |
| ln (per capita GDP) | 1,208 | 8.990 | 1.209 | 6.08 | 11.70 |
| Host | 1,208 | 0.006 | 0.076 | 0 | 1 |
| Ex-Soviet bloc (outside EU) | 1,208 | 0.110 | 0.313 | 0 | 1 |
| Ex-Soviet Bloc (EU member) | 1,208 | 0.032 | 0.177 | 0 | 1 |
| Socialist state (China excluded) | 1,208 | 0.012 | 0.107 | 0 | 1 |
| China | 1,208 | 0.006 | 0.076 | 0 | 1 |
| Schooling | 1,208 | 8.238 | 3.256 | 0.94 | 15.10 |

Table S7. Descriptive statistics (Olympics analysis- final year observations)

|  | Obs | Mean | Std. dev. | Min | Max |
| --- | --- | --- | --- | --- | --- |
|  |  |  |  |  |  |
| Share of medals | 179 | 0.005 | 0.014 | 0.000 | 0.124 |
| ln (population) | 179 | 15.808 | 1.978 | 10.964 | 21.044 |
| ln (per capita GDP) | 179 | 9.204 | 1.152 | 6.535 | 11.648 |
| Host | 179 | 0.006 | 0.075 | 0 | 1 |
| Ex-Soviet bloc (outside EU) | 179 | 0.095 | 0.294 | 0 | 1 |
| Ex-Soviet Bloc (EU member) | 179 | 0.061 | 0.241 | 0 | 1 |
| Socialist state (China excluded) | 179 | 0.011 | 0.105 | 0 | 1 |
| China | 179 | 0.006 | 0.075 | 0 | 1 |
| Schooling | 179 | 9.311 | 2.996 | 2.602 | 15.102 |

Table S8. Olympic Games models. Polychoric correlation matrix

| Variables | (1) | (2) | (3) | (4) | (5) | (6) | (7) | (8) | (9) |
| --- | --- | --- | --- | --- | --- | --- | --- | --- | --- |
| (1) Share of medals | 1.000 |  |  |  |  |  |  |  |  |
| (2) ln(population) | 0.411 | 1.000 |  |  |  |  |  |  |  |
| (3) ln(per capita GDP) | 0.258 | -0.101 | 1.000 |  |  |  |  |  |  |
| (4) Host | 0.272 | 0.518 | 0.257 | 1.000 |  |  |  |  |  |
| (5) Ex-Soviet bloc (outside EU) | 0.145 | 0.046 | 0.008 | -0.898 | 1.000 |  |  |  |  |
| (6) Ex-Soviet Bloc (EU member) | 0.038 | -0.033 | 0.475 | -0.591 | -0.942 | 1.000 |  |  |  |
| (7) Socialist state (China excluded) | -0.349 | 0.266 | -0.335 | -0.843 | -0.915 | -0.649 | 1.000 |  |  |
| (8) China | 0.308 | 0.961 | -0.128 | 0.581 | -0.896 | -0.591 | -0.843 | 1.000 |  |
| (9) Schooling | 0.344 | -0.050 | 0.757 | 0.318 | 0.600 | 0.849 | -0.223 | 0.027 | 1.000 |

Table S9. Schooling levels by World Bank region (Chess Olympiad)

|  | Obs | Mean | Std. dev. | Min | Max |
| --- | --- | --- | --- | --- | --- |
|  |  |  |  |  |  |
| East Asia & Pacific | 173 | 9.50 | 2.46 | 3.30 | 13.40 |
| Europe & Central Asia | 612 | 11.77 | 1.44 | 6.57 | 15.10 |
| Latin America & Caribbean | 266 | 8.25 | 1.69 | 3.66 | 12.39 |
| Middle East & North Africa | 169 | 8.40 | 2.38 | 2.80 | 12.84 |
| North America | 36 | 12.09 | 1.88 | 8.83 | 13.84 |
| South Asia | 64 | 5.28 | 1.84 | 1.70 | 9.16 |
| Sub-Saharan Africa | 199 | 6.24 | 1.91 | 1.12 | 10.19 |
